# Supplementary figures and images for: Non-canonical miRNA-RNA base-pairing impedes tumor suppressor activity of miR-16
Source: Life Sci Alliance. 2022 Oct 6;5(12):e202201643. doi: 10.26508/lsa.202201643 (PMC9553902; doi:10.26508/lsa.202201643)

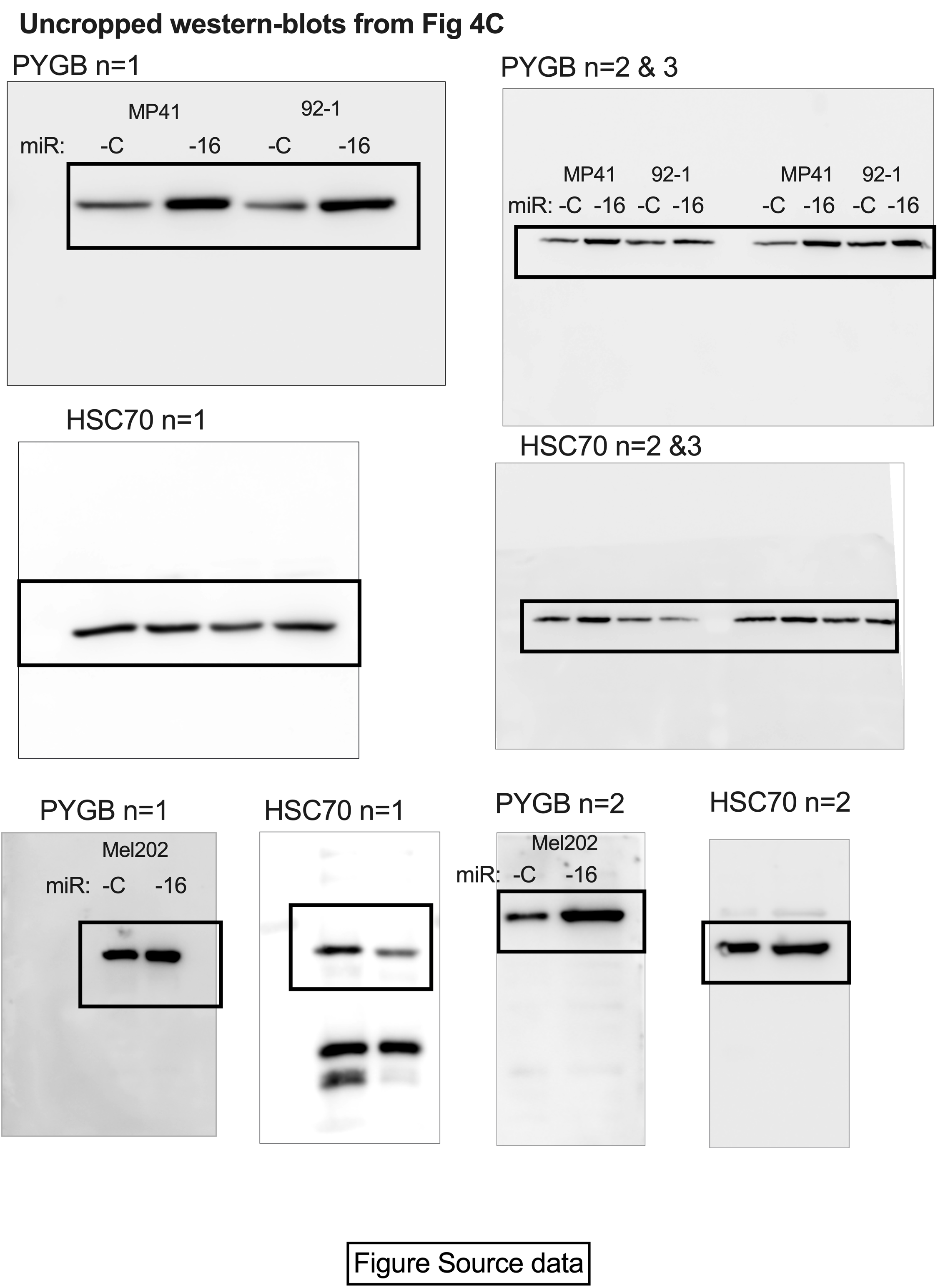

Supplement: Supplementary file 2 [file LSA-2022-01643_SdataF4.tif]
